# The cost of the circadian desynchrony on the Leydig cell function

**DOI:** 10.1038/s41598-022-19889-9

**Published:** 2022-09-15

**Authors:** Maja V. Pavlovic, Dijana Z. Marinkovic, Silvana A. Andric, Tatjana S. Kostic

**Affiliations:** 1 Faculty of Sciences and Mathematics, University of Pristina in Kosovska Mitrovica, 38220 Kosovska Mitrovica, Serbia; 2 Laboratory for Chronobiology and Aging, Department of Biology and Ecology, Faculty of Sciences, University of Novi Sad, Dositeja Obradovica Sq 2, 21000 Novi Sad, Serbia; 3 Laboratory for Reproductive Endocrinology and Signaling, Department of Biology and Ecology, Faculty of Sciences, University of Novi Sad, Dositeja Obradovica Sq 2, 21000 Novi Sad, Serbia

**Keywords:** Reproductive biology, Reproductive disorders

## Abstract

The increased frequency of different lifestyles that disrupts circadian rhythms, together with a trend in the accretion of male idiopathic infertility, imposes the necessity to understand the contribution of circadian rhythms disruption to fertility regulation. In this study, the effects of circadian desynchrony (CD) on the steroidogenic capacity of adult Leydig cells were studied. Adult rats were housed under a disturbing light regime (2 days of constant light, 2 days of continual dark, and 3 days of 12:12 h light:dark schedule) designed to mimic shiftwork in humans. CD was characterized by changed and decreased rhythmic locomotor activity and reduced blood testosterone. In the Leydig cells changed transcription of the clock genes (*Bmal1, Clock, Cry1 and Reverba/b* increased while *Per1/2* reversed phase) was detected. This was followed by reduced transcription of genes (*Star*, *Cyp11a1*, and *Hsd3b1/2*) primarily involved in mitosteroidogenesis. In parallel, mitochondrial membrane potential (Δψi) and ATP production declined losing their characteristic oscillatory pattern. Also, the main markers of mitochondrial biogenesis (*Ppargc1a, Nrf1, Tfam, Cytc*), fusion (*Mfn2*), and mitophagy (*Pink1* and *Tfeb*) were disturbed. Collectively, CD targets mitochondria in Leydig cells by reducing mitosteroidogenesis, mitoenergetics, and disturbing mitochondrial dynamics. These changes contribute to testosterone decline compromising androgen-dependent functions, including reproduction.

## Introduction

The essential condition for the survival of the species is successful and efficient reproduction. This condition can be met if the physiological processes are organized to form a unified system synchronized with the external environment. One of the systems which anticipate daily environmental cycles and thereby enable adaptation and maintenance of dynamic organism's equilibrium is the circadian clock. In mammals, the circadian system is hierarchically organized and composed of the master rhythm regulator located in the suprachiasmatic nucleus of the hypothalamus and peripheral clocks present in peripheral cells^1^. Clock genes orchestrate circadian rhythm in almost every cell operating through transcription/translation negative feedback loop maintaining its rhythm and the rhythmic expression of the clock-dependent genes. The principal genes involved in a clock loops are positive *Bmal1*, *Clock*, *Npas2*, and negative genes *Per1/2/3*, *Cry1/2*, and *Reverba/b*^2^*.*

The circadian clock system, together with neuronal, hormonal, and metabolic signals, collectively regulates reproductive physiology on a 24-h scale and drives diurnal oscillation in gene expression^1^. In males, testosterone needed to complete spermatogenesis and develop and maintain male sexual characteristics is produced by Leydig cells in a daily rhythm. Testosterone is formed by the activity of a battery of steroidogenic elements/enzymes located in mitochondria and endoplasmic reticulum encoded by steroidogenic genes (*Star*, *Cyp11a1*, *Hsd3b1/2*, *Cyp17a1*, *Hsd17b*)^3^. So far, clock genes (*Bmal1*, *Per1/2/3*, *Cry1/2*, *Dbp* and *Reverba/b*), as well as genes involved in the process of steroidogenesis (*Star*, *Cyp11a1*, *Cyp17a1*), have rhythmic expressions in Leydig cells^4–9^. Accordingly, blood testosterone fluctuations usually show one or two peaks^4,10–13^. The generator of these fluctuations is located both in Leydig cells and outside, and steroidogenic function is tuned to regular episodic gonadotropin input^13^. Male Bmal1^−/−^ mice are infertile and have low blood testosterone and decreased expression of testicular steroidogenic genes (*Star*, *Hsd3b2*, and *Hsd17b3*)^14^. Similar results were obtained on BMAL1 knocked out TM3 cells^15^. In contrast, overexpression of BMAL1 significantly increased StAR and HSD17B3 and improved testosterone production^9^. All these observations emphasize the importance of the clock system in maintaining rhythmic testosterone synthesis and, consequently, male fertility.

However, modern lifestyles imposes activity that is not in line with the endogenous rhythm dictated by the circadian clock, leading to so-called “circadian desynchrony” (CD)^16^. This condition can cause changes in hormone secretion, favoring infertility-related changes that may result from reproductive axis dysfunction. As time and synchronization of hormone secretion and tissue sensitivity play a key role in fertility, desynchrony between the master rhythm regulator and the rest of the reproductive axis, especially with peripheral clocks such are in testicular Leydig cells, may become important factors in the development of the conditions associated with reduced testosterone and fertility.

Given the increased frequency of work in shifts and travel that disrupts circadian rhythms, it is crucial to understand the contribution of circadian rhythms to fertility regulation. In particular, understanding the relationship between the circadian clock and testicular function, especially in terms of increased incidence of male infertility^17^ and that the cause of male infertility often remains unknown in over 70% of cases^18^.

Therefore, the current study aims to analyze effect of CD, established by changing of photoperiod, on rat androgen-producing Leydig cells. We hypothesized that CD reduces rhythmic function of the Leydig cells by changing activity of clock and steroidogenesis–related genes.

## Results

Decreased male fertility is a growing health problem that requires a better understanding of molecular events regulating reproductive competence. Although previously published data suggest an association between circadian rhythm disruption and decreased male fertility^19^, the knowledge about how CD interrupts male fertility is still incomplete. In this study, the effects of CD on the androgenic capacity of adult Leydig cells were studied. Adult rats were housed under a disturbing light regime (2 days of constant light, 2 days of continual dark, and 3 days of 12:12 h light:dark schedule) designed to mimic shiftwork in humans. After 2 months of life in conditions of the altered light regime, the transcriptional pattern of genes essential for rhythmic Leydig cell's endocrine activity and blood hormones level was monitored at five different time points.

### Effects on rhythmic daily activity and blood hormones

**Figure 1:**
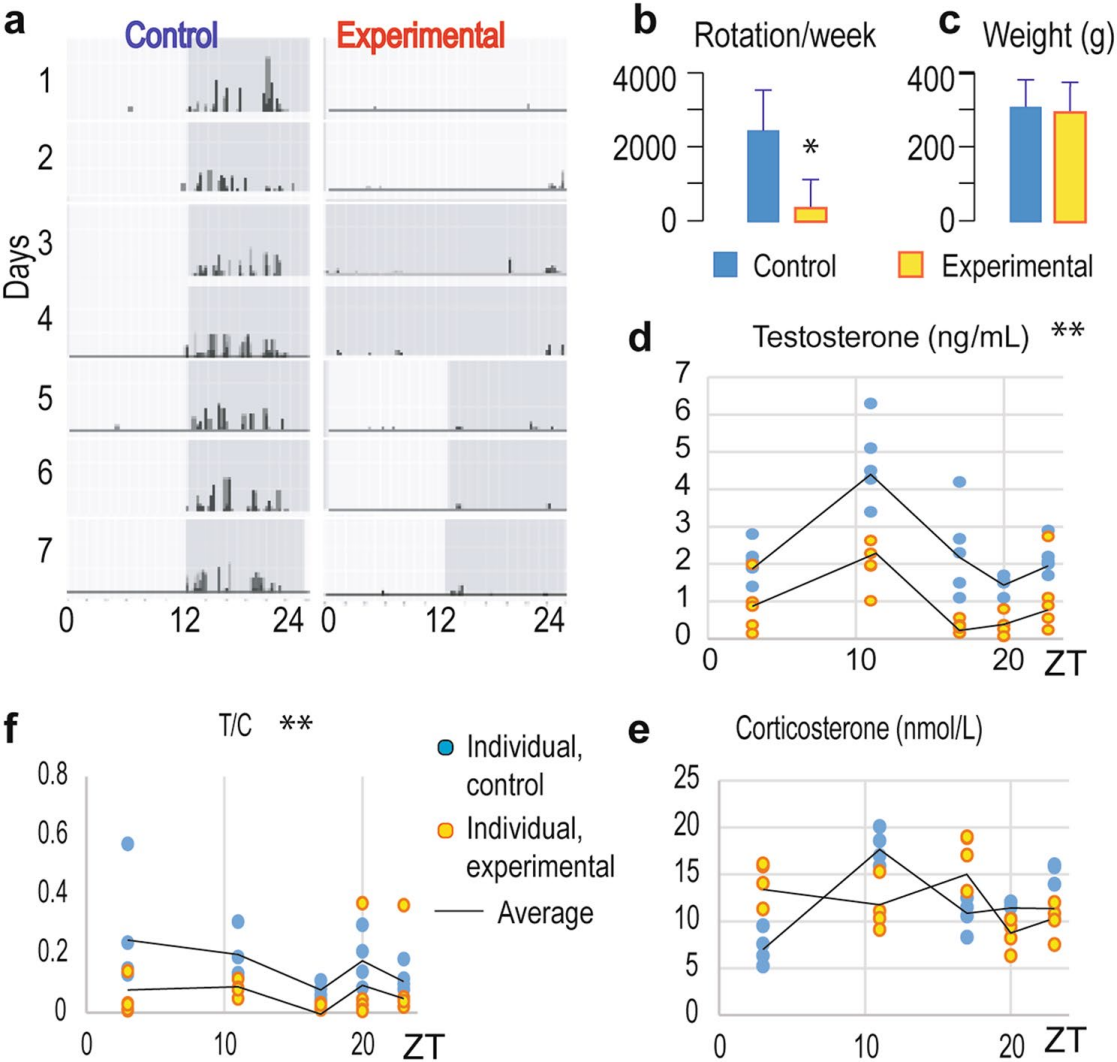
The CD changed daily activity pattern and reduced testosterone blood level. Adult rats were housed under the controlled light regime of 12 h light–12 h dark (control) or were exposed to a disturbing light regime (2 days of continual light, 2 days of continual dark, and 3 days of 12:12 h light:dark schedule; experimental) for 2 months. The voluntary activity was monitored, and actograms were formed. The representative weekly actogram from control and experimental rats (**a**) as well as average weekly activity (**b**) and body mass (**c**) in the last week of the experiment are shown in bars ± SEM values (n = 25; 5 rats in 5 time points). The experimental conditions reduced diurnal fluctuations of serum testosterone (determined in ZT3, ZT11, ZT17, ZT20, ZT23; ZT0 is time when light switch on) (**d**), flattened the daily profile of corticosterone levels in serum (**e**), and reduced testosterone/corticosterone (T/C) ratio (**f**). For rhythm parameters, please see Suppl. Table 2. ZT is zeitgeber time, i.e., the time when the light switches on. In (**d**–**f**) panels of this figure and all others figures data points represent individual values, while the black line represents group means values (n = 5). **Statistical significance p < 0.01 determined by two-way ANOVA; *statistical significance p < 0.05 determined by Mann–Whitney test.

The CD was characterized by decreased and arrhythmic voluntary rat activity (Fig. 1a; Supplemental Fig. 1). It seems that the rats' activity in the experimental group decreased with time (Supplemental Fig. 1) so that in the last week of the experiment, it was about six times lower in the experimental compared to the control group (Fig. 1b). However, the body weight was not changed (Fig. 1c) during 2 months of experiment. In the blood of control rats, 24 h fluctuations of testosterone and corticosterone levels were observed with a peak around ZT11 for both (Fig. 1d,e; Suppl. Table 2). CD disturbed testosterone's diurnal rhythm in blood by decreasing mesor and amplitude (Fig. 1d; Suppl. Table 2) but peak phasing was maintained. To assess stress levels in CD rats' serum corticosterone was measured. No overall increase in diurnal corticosterone blood levels was observed, but the characteristic pattern of blood corticosterone diurnal variation was lost (Fig. 1e; Suppl. Table 2). However relative ratio between testosterone and corticosterone normalized on value ZT3 in control samples (T/C ZT3 = 1) decreased in CD condition (Fig. 1f) suggesting T/C as a possible biomarker for changes in behavior in response to circadian desynchrony.

**Figure 2:**
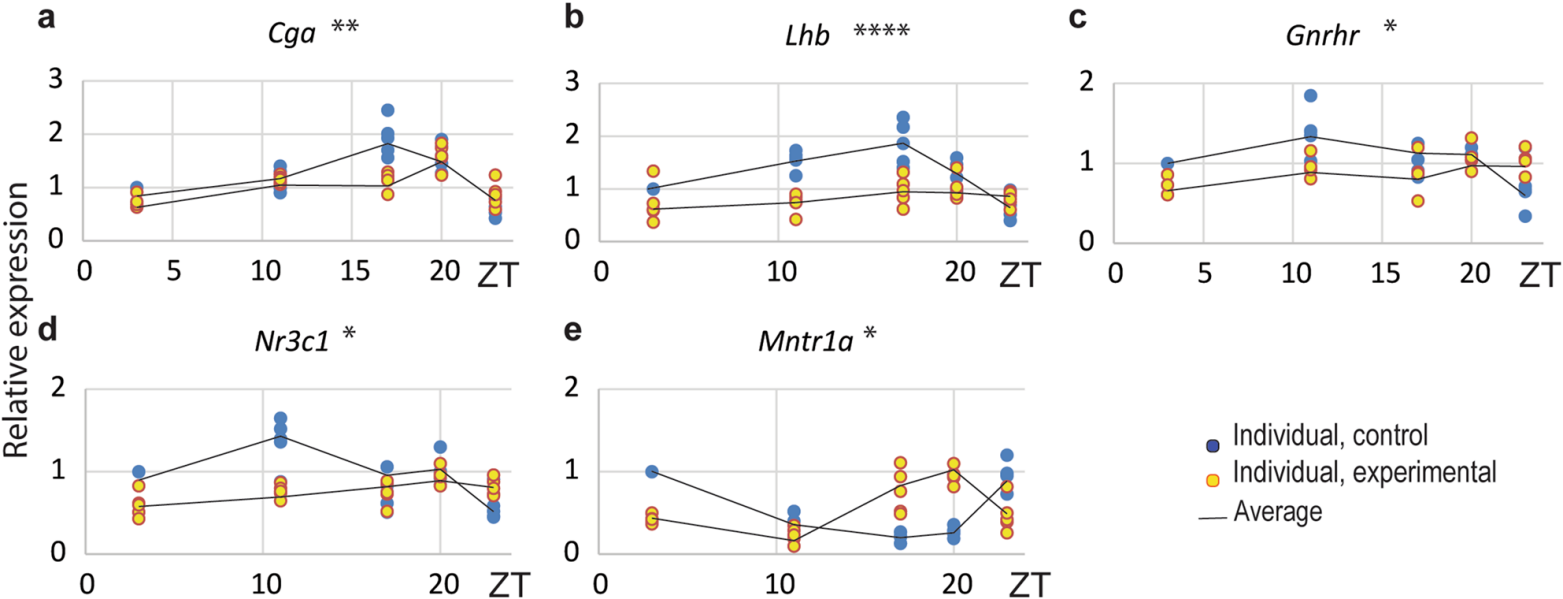
The CD changed daily expression patterns of the pituitary genes. Pituitary were collected at different time points during 24 h, RNA isolated for RT followed by RQ-PCR analysis of genes encoded LH subunits (**a**,**b**) and genes encoding receptors essential for gonadotroph function (**c**–**e**). CD changed daily transcriptional pattern of *Cga* (**a**), *Lhb* (**b**), *Gnrhr* (**c**), *Nr3c1* (**d**) and *Mntr1*a (**e**). Statistical significance: *p < 0.05; **p < 0.01; ****p < 0.001 determined by two-way ANOVA.

Further, since luteinizing hormone (LH) is the primary regulator of Leydig cell steroidogenesis, the transcription of pituitary genes encoding LH subunits (*Lhb*, *Cga*) and genes encoding receptors essential for gonadotroph function (*Gnrhr*, *Nr3c1*, *Mntr1a* which encrypt gonadotropin releasing hormone receptor, glucocorticoid receptor, and melatonin receptor, respectively) were studied. As expected, the result showed the diurnal variation of *Cga* (Fig. 2a; Suppl. Table 2) and *Lhb* (Fig. 2b; Suppl. Table 2) but also *Gnrhr* (Fig. 2c; Suppl. Table 2), *Nr3c1* (Fig. 2d; Suppl. Table 2) and *Mntr1a* (Fig. 2e; Suppl. Table 2) proving circadian activity of pituitary genes involved in the regulation of reproduction. Furthermore, CD disturbed a daily expression profile of *Cga* and *Lhb* (Fig. 2a,b; Suppl. Table 2), suggesting a possible disturbance and decrease in LH synthesis that, in addition to changed transcriptional pattern of *Gnrhr* (Fig. 2c), *Nr3c1* (Fig. 2d) and *Mntr1a* (Fig. 2e) propose disorder in pituitary rhythmic activity, at least the one related to the regulation of reproduction.

### Expression of the genes related to rhythmic endocrine Leydig cell function

**Figure 3:**
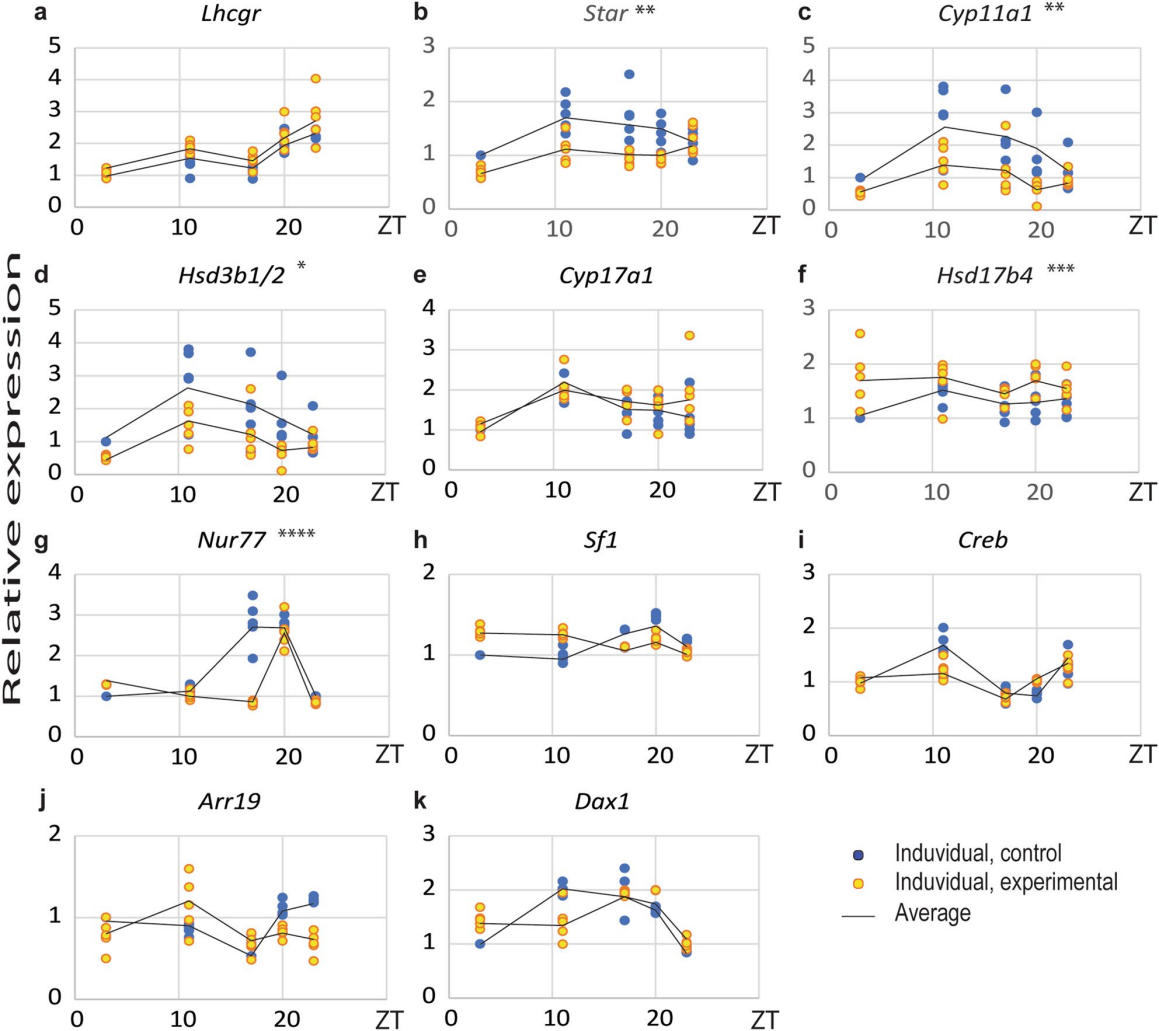
CD changed expression of the genes principally related to mitosteroidogenesis. Leydig cells from both groups were individually isolated at different time points for 24 h. RQ-PCR analysis was performed on steroidogenesis-related genes (**a**–**f**) and main transcription factors regulating steroidogenic elements expression (**g**–**k**). CD affected the transcription pattern of genes involved in steroidogenesis in mitochondria (**b**–**d**) but also positive (*Nur77*, **g**) and negative regulatory genes (*Arr19*, **j**). Statistical significance: *p < 0.05; **p < 0.01; ***p < 0.005; ****p < 0.001 determined by two-way ANOVA.

Since CD effectively decreased blood testosterone levels, the transcription of genes encoding the main elements essential for steroidogenesis was analyzed in the Leydig cells. The analysis was performed on the second day of the continual light phase in experimental and at the same time in the control groups. Obtained data in controls confirmed daily fluctuation of the transcription of key steroidogenic genes, *Star*, *Cyp11a1*, and *Cyp17a1* (Fig. 3b,c,e; Suppl. Table 2) while *Lhcgr* (Fig. 3a) which determines Leydig cells' sensitivity on LH, and *Hsd3b1/2* (Fig. 3d) did not show 24 h variation^4,13^. CD reduced mesor and amplitude of the *Star* and *Cyp11a1* (Fig. 3b,c; Suppl. Table 2). These genes encode enzymes that are functionally linked to mitochondria, proposing that CD target mitosteroidogenesis. However, overall diurnal rhythmicity and peak phasing of these gene transcripts in the Leydig cells was maintained (Suppl. Table 2). CD also decreased the non-cyclical expression of *Hsd3b1/2* and increased the *Hsd17b4* (Fig. 3d,f), suggesting that treatments in addition to mitochondrial part also targets the final step of testosterone production in Leydig cells.

Next, the gene expression of the most important transcription factors that regulate steroidogenesis was performed (Fig. 3g–k; Suppl. Table 2). All analyzed transcription factors, except *Sf1* (Fig. 3h) showed daily fluctuation in its activity: positive regulators, *Nur77* (Fig. 3g; Suppl. Table 2) peaked in dark (active) while *Creb* (Fig. 3i; Suppl. Table 2) reached a maximum at the end of light (inactive) phase; negative regulators *Dax1* (Fig. 3k; Suppl. Table 2) peaked at the beginning of dark while *Arr19* (Fig. 3j) at the beginning of light period. In CD conditions expression pattern of *Sf1, Creb*, *Dax1* and *Arr19* (Fig. 3h–k; Suppl. Table 2) were preserved. However, mRNA for *Nur77* was downregulated at ZT17 (Fig. 3g).

**Figure 4:**
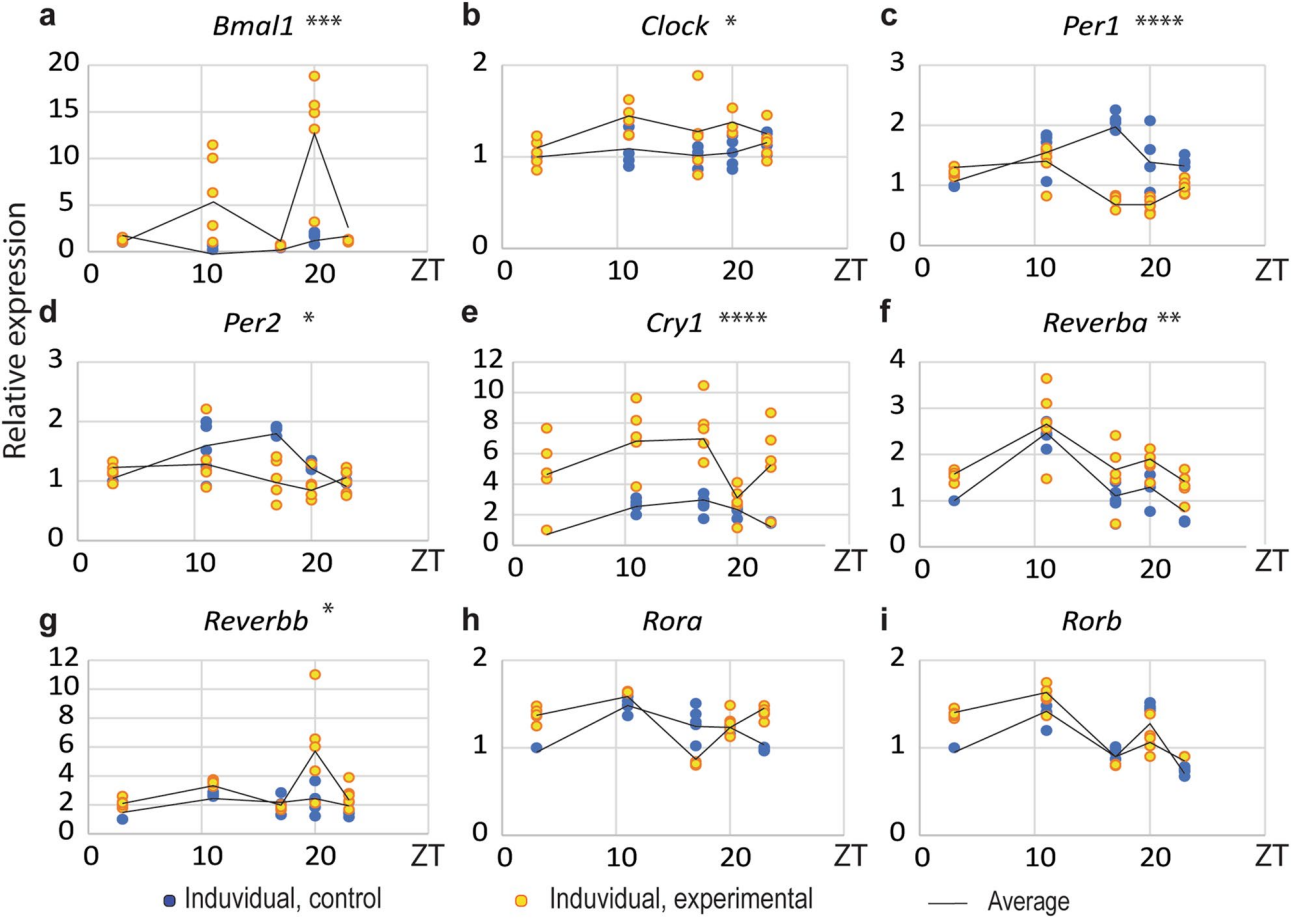
CD changed clock genes expression. Leydig cells from both groups were individually isolated at different time points for 24 h. RQ-PCR analysis of clock genes were performed in the second day of the light period in the CD protocol. CD changed diurnal transcriptional rhythms: increased positive regulators *Bmal1* (**a**) and *Clock* (**b**) and negative *Cry1* (**e**), *Reverba* (**f**) and *Reverbb* (**g**) but also decreased *Per1* (**c**) and *Per2* (**d**) in ZT11 and ZT17. Transcription of *Rora* (**h**) and *Rorb* (**i**) were unaltered. Statistical significance: *p < 0.05; **p < 0.01; ***p < 0.005; ****p < 0.001 determined by two-way ANOVA.

In addition, a diurnal clock gene expression profile was measured (Fig. 4). A marked change in diurnal transcription rhythms was observed for: positive clock elements *Bmal1* (Fig. 4a) and *Clock* (Fig. 4b), and negative *Cry1* (Fig. 4e), *Reverba* (Fig. 4f), and *Reverbb* (Fig. 4g) which have increased without changes in expression times (Suppl. Table 2) while *Per1* (Fig. 4c) and *Per2* (Fig. 4d) reversed phase. Positive regulators within the secondary loop, *Rora* (Fig. 4h) and *Rorb* (Fig. 4i), substantially did not change transcription rhythm.

### CD changed mitochondrial function in Leydig cells

**Figure 5:**
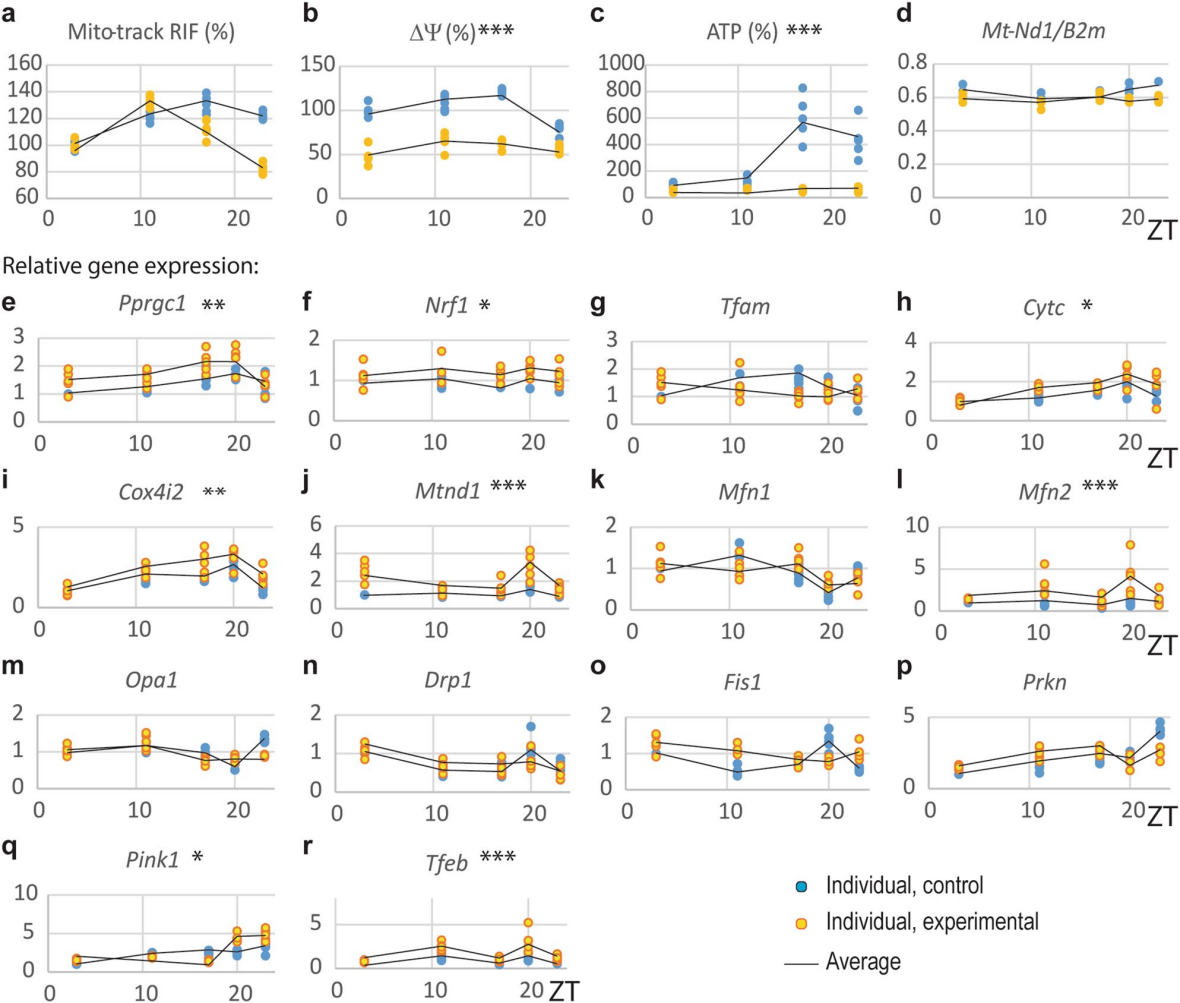
CD changed mitochondrial function. Leydig cells from both groups were individually isolated at different time points during 24 h and followed by measurements to estimate mitochondrial mass and function: mitochondrial mass was estimated through mitotrack assay (**a**) and *Mt-Nd1*/*B2m* ratio (**d**), mitochondrial membrane potential (ΔΨ) was estimated through TMRE measurement (**b**) and mitochondrial energetic function was estimate through ATP measurement (**c**). CD decreased mesor of ΔΨ and ATP production. Mitochondrial biogenesis were estimated by transcriptional activity for several genes (**e**–**j**); mitochondrial fusion (**k**–**m**); mitochondrial fission (**n**,**o**) and mitophagy (**p**–**r**). Statistical significance: *p < 0.05; **p < 0.01; ***p < 0.005; ****p < 0.001 determined by two-way ANOVA.

Because the expression of genes involved in mitosteroidogenesis was decreased, it was reasonable to investigate other aspects of mitochondrial physiology, especially energetic function. Since mitochondrial membrane potential (Δψm) contributes to many processes, including mitochondrial energetics and steroidogenesis^20^, it was interesting to examine whether CD affects the Δψm value in Leydig cells. Changes in the electrochemical gradient Δψm were measured by TMRE fluorescence since the magnitude of TMRE fluorescence is proportional to the Δψm changes. Obtained results revealed daily rhythmic variations of the Δψm in Leydig cells, which reaches a maximum at the end of the light and the beginning of the dark phase (Fig. 5b) and coincides with the time the increase in blood testosterone (Fig. 1d). Also, diurnal rhythmicity of ATP values was observed in Leydig cells from control rats (Fig. 5c; Suppl. Table 2). ATP reached its maximum in the dark phase (Fig. 5c; Suppl. Table 2) close to the peak of the Δψ. However, CD decreased mesor of Δψm fluctuation compared to control groups without altering peak phasing (Fig. 5b) and reduced and flattened the pattern of ATP oscillation in Leydig cells (Fig. 5c; Suppl. Table 2). The observed decrease of Δψm and ATP in CD rats was not associated with the overall decrease of mitochondrial mass estimated through *Mt-Nd1*/*B2m* ratio (Fig. 5d) or mitotrack assay (Fig. 5a).

It is well known that mitochondrial function is regulated by mitochondrial fusion/fission closely linked with mitobiogenesis and mitophagy^21^. To see if CD in Leydig cells is associated with changes in mitochondrial fusion/fission or/and by altered mitochondrial biogenesis or mitophagy the expression of genes involved in these processes were monitored by RQ-PCR. An upregulation of mRNA levels in CD conditions were observed for *Ppargc1a* (main regulator of mitochondrial biogenesis and function; Fig. 5e), its downstream gene *Nrf1* (Fig. 5f) as well as *Cytc* (Fig. 5h), *Cox4* (a subunit of complex IV; Fig. 5i) and *Mtnd1* from mitochondrial genome (Fig. 5j). However, transcriptional pattern of *Tfam* (Fig. 5g) was not significantly altered. Among mitochondrial profusion genes, only *Mfn2* (Fig. 5l) was stimulated while *Mfn1* (Fig. 5k) and *Opa1* (Fig. 5m) were unchanged. Transcription of profission genes *Drp1* (Fig. 5n) and *Fis1* (Fig. 5o) was without changes in CD group while *Pink1* (Fig. 5q) and *Tfeb* (Fig. 5r) were up regulated but not *Prkn* (Fig. 5p). Obtained results proposed activation of genes responsible for mitochondrial biogenesis but also activation of genes that promote mitophagy, establishing an equilibrium in the relationship of these processes without changing the mass but with decreased mitochondrial functionality.

## Discussion

Infertility or reduced fertility is a global health problem that affects 8–12% of couples worldwide^22^. Male infertility accounts for about 50% of detected cases, of which for almost 70%, the cause is unknown^18^. Previous pre-/clinical studies suggested that CD may negatively interfere with blood levels of reproductive hormones and sperm function^19,23–25^. However, the pieces of evidence are still debatable, especially in terms of specific processes that are affected by CD. Therefore, we analyzed the effects of CD, induced by changed photoperiodism, on adult rat Leydig cell testosterone production.

Our data indicate that CD changed rhythmic Leydig cells activity leading to decreased testosterone synthesis. This is associated with altered rhythmic transcription of core clock genes (increased *Bmal1*, *Clock*, *Cry1*, *Reverba/b* and reversed pattern of *Per1/2*) and steroidogenic genes (reduced *Star*, *Cyp11a1*, *Hsd3b1/2* and *Hsd17b4*). Reduced testosterone production and observed changes in genes expression are not the results of stress since the elevation of blood corticosterone secretion was not detected. Opposite, in CD caused by inverted feeding, high circulating levels of corticosterone were observed^26^, indicating cooperation of clock and stress systems in response to changes in the environment. On the contrary, our experimental CD model is driven by a mismatch of endogenous circadian rhythms with altered weekly photoperiodic cycles, and adaptation to newly emerging conditions does not include a stress response. Yet, disturbed daily transcriptional profiles of genes encoding LH and GNRHR were observed, suggesting disturbance of pituitary rhythmic activity. Since a temporally organized and synchronized reproductive axis is essential for reproduction^27^, pituitary and Leydig cells' rhythm disorders could be related to a decrease in testosterone synthesis and weakening of reproductive function.

The obtained results showed that CD alters the endocrine function of Leydig cells by disrupting mitochondrial function. This is supported by three lines of evidences indicating reduced mitosteroidogenesis, reduced mitoenergetics, and altered mitochondrial dynamics. First, CD changed the expression of essential steroidogenic genes functionally connected to mitochondria (*Star*, *Cyp11a1*, and *Hsd3b1/2*). The Leydig cell`s mitosteroidogenesis is triggered by LH-cAMP-PRKA signaling, which activates mitochondria-targeted StAR protein involved in cholesterol transport into the inner mitochondrial membrane^28^ and its conversion to pregnenolone by CYP11A1 followed by conversion to progesterone by HSD3b1/2^29^. Activation of mitochondrial CYP11A1 is a hormonally regulated and rate-limiting step and is considered as a determinant of the steroidogenic capacity of the cells^30^. Besides, CD abolishes the characteristic rhythmic transcriptional profile of the *Nur77* that regulates expression of steroidogenesis-related genes, including *Star* and *Hsd3b1/2*^31^.

Second, CD changed the diurnal profile and dampened ΔΨm fluctuation and ATP production in Leydig cells. It is known that maintenance of ∆ψm is critical for ATP synthesis and steroidogenic mitochondrial activities^20,32–34^. Actually, mitochondrial electron transport chain, which drives formation of the ∆ψm utilized for ATP synthesis, also facilitate cholesterol transport into mitochondria and LH-mediated testosterone production^20^. In Leydig cells from control rats, these processes are temporally synchronized on 24 h scale because the peak of ∆ψm fluctuations occurs at the end of the light phase close to the height of blood testosterone, followed by a maximum of ATP production. Further, gonadotropin stimulation of Leydig cells provokes increase of ∆ψm, ATP and testosterone production^34^ suggesting possible synchronization through hormones of reproductive axis.

Third, CD changed the transcription of main markers of mitochondrial biogenesis and mitophagy. It is well known that the effective energetic and steroidogenic cell function is determined by the quantity and dynamics of the mitochondrial network regulated by specific gene activities^21,35^. In Leydig cells from CD rats, the increased transcription of main markers of mitochondrial biogenesis (*Ppargc1a*, *Nrf1*, *Cytc*, *Cox4*, and *Mtnd1* from the mitochondrial genome) was detected, suggesting a possible effect on mitochondrial mass. However, mitochondrial content, as measured by levels of mtDNA, or Mitotrack assay, was not found to be changed, implying increased mitochondrial removal through mitophagy. Such a prediction is supported by the up-regulation of *Pink1* and *Tfeb*, which are involved in maintaining mitochondrial homeostasis through mitophagy. Still, a lot of data shows circadian clock involvement in regulating mitochondrial biogenesis, fission/fusion, and mitophagy^36^. For example, the CLOCK-BMAL1 complex stimulates mitochondrial biogenesis and mitophagy by activating SIRT1^36^. A similar scenario may occur in Leydig cells in circadian desynchrony when *Bmal1* and *Clock* are stimulated but also genes regulating mitochondrial biogenesis (*Ppargc1a*, *Nrf1*) and mitophagy (*Pink1* and *Tfeb*). Additionally, most of the genes (*Ppargc1a*, *Cytc*, *Cox4*, *Tfam*, *Mfn1/2*, *Drp*, *Pink1*, and *Parkin*) are involved in the regulation mitobiogenesis/dynamics/mitophagy in Leydig cells show daily fluctuation in transcriptional activity, but again, without detected effect on mitochondrial mass. This finding is in line with observation in human skeletal muscle^37^ and in synchronized immortalized human hepatic cells^38^, supporting the role of the circadian clock orchestrating the functioning of mitochondria which, therefore, may be impaired in circadian desynchrony.

In addition to affecting the Leydig cell's mitochondrial function, the CD also targets the final step in testosterone production by stimulating *Hsd17b* expression. The *Hsd17b* encodes the enzyme responsible for the last and critical step in testosterone synthesis, i.e., converting androstenedione to testosterone^39^. Therefore, an increase in transcription of *Hsd17b* may be associated with an increase in *Bmal1* since it was shown that BMAL1 positively regulates *Hsd17b* transcription in goat Leydig cells^9^. However, stimulation of the last step in steroidogenesis is not enough to overcome the adverse effects of CD on testosterone production.

**Figure 6:**
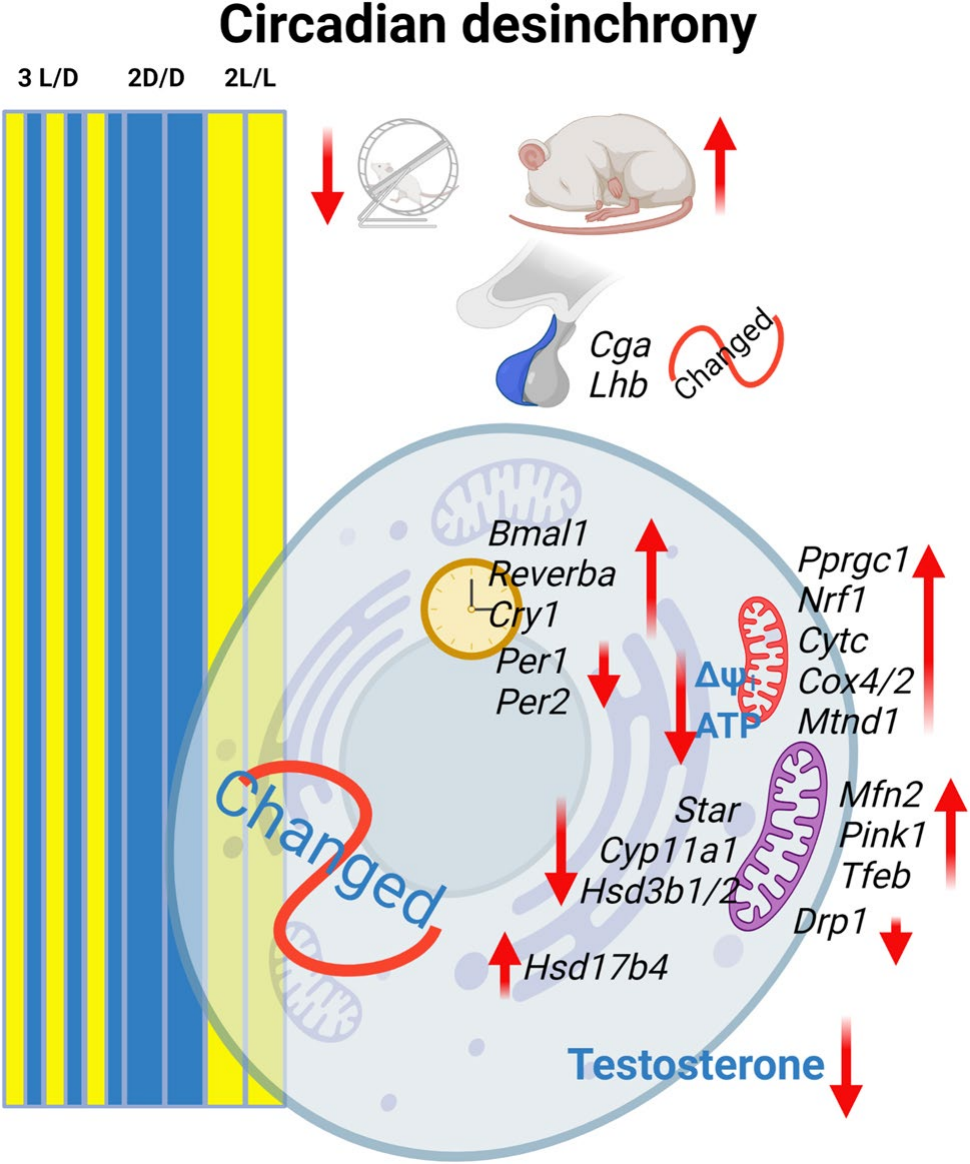
The cost of the circadian desynchrony on the Leydig cell function. The figure represents processes and genes affected by CD. The figure was created with BioRender.com.

In conclusion, our study reveals the harmful effect of circadian desynchrony established by changing photoperiod on rat Leydig cells physiology. In such conditions, Leydig cells' cost is a decrease in steroidogenic ability, mainly due to a clock disturbance and reduction of mitochondrial function (Fig. 6). Given the importance of testosterone in reproduction, future research is needed to determine the cost of circadian desynchrony on reproductive capacity, including effects on spermatogenesis and sperm functionality.

## Methods

### Ethical approval

Animal maintenance and conduction of all the experiments were performed in the Laboratory of Chronobiology and Aging and the Laboratory for Reproductive Endocrinology and Signaling, Department of Biology and Ecology, Faculty of Sciences, University of Novi Sad. All the experiments and protocols were approved by the Committee on Animal Care University of Novi Sad (statement no. 2020-01-02), operating under the rules of the National Council for Animal Welfare and following statements of National Law for Animal Welfare (copyright March 2009). Every experiment was performed and conducted followed by “European Convention for the Protection of Vertebrate Animals used for Scientific Purposes” (Council of Europe No 123, Strasbourg, 1985) and NIH Guide for the Use and Care of Laboratory Animals (NIH Publications, No. 80 23, revised, 7th ed., 1996). Experiments were in adherence to the ARRIVE guidelines.

### Animals and in vivo experiment

All the experiments were carried out on 9 months old male *Wistar* rats bred and raised in the animal facility of Faculty of Sciences, University of Novi Sad (Serbia). Rodents were grown in controlled temperature conditions (22 ± 2 °C) with food and water ad libitum. As for the light regime, experimental animals were living under 2 days constant light, 2 days constant darkness and 3 days 12 h light and 12 h dark respectively, for 2 months. Control group of animals were held under LD regime. After 2 months, animals were quickly sacrificed by decapitation in five different time points during 24 h (ZT3, ZT11, ZT17, ZT20, ZT23; ZT0 is time when light switch on; five animals per time point for each group). The method of sacrificing was chosen to avoid the effect of anesthesia on blood hormone levels. The experiment was completed on the second day of the light period in the CD protocol. Rats’ body weight was measured once per week during experiment. Afterwards, trunk blood was collected individually, testes were removed for Leydig cells isolation and purification.

### Detection of an animal’s voluntary activity

In order to monitor rat’s voluntary activity, a group of control and experimental rats were placed individually in cages with running wheel system. The detection system was programmed to record turns of the wheel that animal made every 6 min. Activity pattern of rats was monitored for 2 months and data was collected and used for forming actograms as one of the standard ways to represent circadian rhythms. The graphical representation of the animal’s activity was formed using R software.

### Hormone level measurement

Hormone levels were measured by radioimmunoassay. According to anti-testosterone No. 250 100% cross-reactivity with DHT^40^, testosterone level was referred as testosterone + dihydrotestosterone. Samples were measured in duplicate (sensitivity: 6 pg per tube; intraassay coefficient of variation 5–8%). Corticosterone EIA Kit (Caymanchem, Michigan, USA) was used for detecting corticosterone levels, with 30 pg/ml as the lowest standard significantly different from blank. All samples were measured in duplicate.

### Leydig cells purification

Testes were decapsulated, placed in 50 ml plastic tubes (per animal) containing 3 ml of collagenase solution (1.25 mg/ml collagenase; 1.5% bovine serum albumin (BSA); 20 mM HEPES, Sigma, St Louise, Missouri) and incubated in shaking-water bath (15 min/34 °C/120 cycles/min). The collagenase reaction was stopped by adding cold M199 medium and seminiferous tubules were removed by filtration. The resulting interstitial cells’ suspension was centrifuged at 160×*g*/5 min. Afterwards, cell pellet was resuspended (8 ml per animal) using DMEM/F12. Resuspended cells were placed on Percoll gradients (Sigma, St Louis, Missouri) with densities of 1.080, 1.065 and 1.045 g/ml and centrifuged 1100×*g*/28 min (without brake). Purified Leydig cells were collected from 1.080/1.065 g/ml and 1.065/1.045 g/ml and washed with adequate amount of 0.1% BSA-M199, centrifuged at 200×*g*/5 min and resuspended in 5 ml DMEM/F12. According to HSD3B staining^3^ proportion of Leydig cells presence was around 90%, and viability of the cells according to the Trypan blue exclusion test was greater than 90%. The suspension of Leydig cells was further centrifuged and the pellet was stored at − 70 °C until RNA and protein analysis.

### Mitochondrial abundance and mitochondrial membrane potential

After Leydig cell isolation, as previously described, cells were plated in two 96 well-plate (1 × 10^5^ and 0.5 × 10^5^ cells/well) and incubated with tetramethylrhodamine (TMRE; Thermo Fisher Scientific, Waltham, MA, USA) and MitoTracker Green, respectively, staining for 20 min/34 °C/5% CO_2_. Fluorescence was detected on fluorimeter (Fluoroscan, Ascent, FL) using excitation wavelengths 590 and 510 nm whereas emission wavelengths 550 and 485 nm, respectively. Cells were washed using 0.1% BSA-PBS and stored until Bradford method protein quantification.

### ATP level measurement

Level of ATP was estimated using the ATP Bioluminescence CLS II kit (Roche Diagnostics, Mannheim, Germany) following the protocol recommended by the manufacturer. Leydig cells (1 × 10^6^/tube) were resuspended in boiling water and Tris–EDTA (1:9), incubated 3 min/100 °C and centrifuged 900×*g*/1 min. The resulting supernatant was used for ATP level detection whilst the pellet was further utilized for Bradford method analysis. Luciferase reagent was mixed together with standard/sample (1:1) in order to measure luminescence (Biosystems/luminometer, Fluorescan, Ascent, FL).

### Genomic DNA isolation, total RNK purification, RT-PCR and RQ-PCR analysis

Genomic DNA from Leydig cells was purified using NucleoSpin Tissue, DNK, RNA and protein purification Macherey-Nagel (Dueren, Germany). Total RNA from Leydig cells and pituitary gland was isolated using GenEluteTM Mammalian Total RNA Miniprep (Qiagen, Hilden, Germany) and EXTRAzol reagent (Birt, Gdańsk, Poland) respectively, following a recommended protocol. DNA and RNA quality was measured and validated using BioSpec-nano (Shimadzu Biotech, Kyoto, Japan). Afterwards, RNA samples were treated with DNase-I treatment (New England Biolabs, Ipswich, Massachusetts, USA). cDNA synthesis was performed using HighCapacity kit (Thermo Fisher Scientific, Waltham, MA, USA) according to manufacturer’s protocol. RQ-PRC SYBR-Green based technology (Applied Biosystems/Thermo Fisher Scientific, Massachusetts, USA) was utilized to determine relative gene expression. The transcription of *Gapdh* was used to correct the variations in cDNA between the samples. The reaction was performed in presence of 5 µl cDNA and specific primers (Suppl. Table 1).

### Statistical analysis

Statistical analysis was performed using GraphPad Prism 8. Experimental results are individually presented as scaterogram or shown as mean value ± SEM variation. Two-way ANOVA was used to analyze results between the groups including all time points. Rhythm parameters (p, MESOR, Amplitude and Acrophase) were obtained using cosinor method online, fitted to 24 h (https://cosinor.online/app/cosinor.php) (Suppl. Table 2).

## Supplementary Information

Supplementary Table 1.

Supplementary Table 2.

Supplementary Figure 1.

## Data Availability

The datasets generated and analyzed during the current study are available in the Cloud of Faculty of Sciences University of Novi Sad, [https://cloud.pmf.uns.ac.rs/s/jDsJS6GwFH6Hrzg]; data from supplemental Fig. 1 are available at [https://cloud.pmf.uns.ac.rs/s/nmcL6idHTDieATy].
