# Supplementary material for: The cost of the circadian desynchrony on the Leydig cell function
Source: Sci Rep. 2022 Sep 15;12:15520. doi: 10.1038/s41598-022-19889-9 (PMC9478133; doi:10.1038/s41598-022-19889-9)
Supplement: Supplementary file 2 — Supplementary Table 2. [file 41598_2022_19889_MOESM2_ESM.docx]

**Supplemental table 2. Rhythm parameters in Leydig cells from control and experimental rats (period fitted to 24h).** Control (L/D) and experimental (2+2+3) rats were euthanized in five time points during the 24h (ZT3, ZT11, ZT17, ZT20 and ZT23). Serum was collected for determination of hormone levels (testosterone and corticosterone); Leydig cells were isolated and used for further analysis (TMRE, MitoTrack, ATP, RQPCR). Presented rhythm parameters were obtained by Cosinor method.

**LEYDIG CELLS**

|  | **Group** | **p** | **Mesor** | **Amplitude** | **Acrophase (ZT)** | |
| --- | --- | --- | --- | --- | --- | --- |
| ***Testosterone*** | L/D | 0.00003 | 2.95710 | 1.54409206 | 11h 01 min | |
|  | 2+2+3 | 0.00729 | 1.14191 | 0.74927550 | 8h 13 min | |
| ***Corticosterone*** | L/D | 0.0505 | 12.9620 | 3.37835408 | 11h 18 min | |
|  | 2+2+3 | 0.95567 | 12.4357 | 0.90599073 | 11h 33 min | |
| ***ΔΨm*** | L/D | 0.00213 | 103.368 | 14.87882998 | 11h 33 min | |
|  | 2+2+3 | 0.01761 | 58.1669 | 7.51210107 | 14h 30 min | |
| ***MitoTrack*** | L/D | 0.00001 | 118.117 | 16.04978225 | 17h 1 min | |
|  | 2+2+3 | 0.00001 | 109.872 | 24.50750541 | 11h 4 min | |
| ***ATP*** | L/D | 0.00001 | 284.530 | 351.1389623 | 18h 48 min | |
|  | 2+2+3 | 0.44046 | 55.5313 | 4.60023274 | 13h 57 min | |
| ***Cga*** | L/D | 0.00018 | 1.19494 | 0.55673667 | 15h 52 min | |
|  | 2+2+3 | 0.00164 | 1.04180 | 0.33512673 | 16h 33 min | |
| ***LHb*** | L/D | 0.00001 | 1.29714 | 0.53724673 | 13h 43 min | |
|  | 2+2+3 | 0.06608 | 0.81198 | 0.18108336 | 18h 21 min | |
| ***Gnrhr*** | L/D | 0.00135 | 1.11977 | 0.27666657 | 11h 16 min | |
|  | 2+2+3 | 0.17831 | 0.91007 | 0.11037424 | 18h 16 min | |
| ***Nr3c1*** | L/D | 0.00145 | 1.03081 | 0.31148932 | 9h 37 min | |
|  | 2+2+3 | 0.0234 | 0.74622 | 0.13215627 | 18h 16 min | |
| ***Mntr1a*** | L/D | 0.00001 | 0.60140 | 0.45777120 | 3h 14 min | |
|  | 2+2+3 | 0.00005 | 0.47423 | 0.35329539 | 19h 15 min | |
| ***Star*** | L/D | 0.00017 | 1.45287 | 0.42599641 | 14h 1 min |  |
|  | 2+2+3 | 0.68424 | 0.98271 | 0.07661131 | 17h 15 min |  |
| ***Lhcgr*** | L/D | 0.18636 | 1.44141 | 0.41309984 | 21h 8 min |  |
|  | 2+2+3 | 0.31333 | 1.81263 | 0.51137684 | 20h 44 min |  |
| ***Insl3*** | L/D | 0.69545 | 1.05808 | 0.08125327 | 20h 51 min |  |
|  | 2+2+3 | 0.73348 | 1.18016 | 0.14121456 | 17h 30 min |  |
| ***Cyp11a1*** | L/D | 0.04785 | 1.49672 | 0.71556807 | 11 h 44 min |  |
|  | 2+2+3 | 0.0571 | 1.04365 | 0.44641311 | 12h 32 min |  |
| ***Cyp17a1*** | L/D | 0.02598 | 1.49049 | 0.38068744 | 12h 38 min |  |
|  | 2+2+3 | 0.45952 | 1.67905 | 0.2583293 | 14h 00 min |  |
| ***Hsd3b1/2*** | L/D | 0.14487 | 1.45042 | 0.38319678 | 15h 45 min |  |
|  | 2+2+3 | 0.16403 | 1.23935 | 0.58720327 | 22h 00 min |  |
| ***Hsd11b1*** | L/D | 0.00009 | 2.5362 | 2.18904729 | 18h 31 min |  |
|  | 2+2+3 | 0.00093 | 2.41411 | 1.55163934 | 19h 18 min |  |
| ***Hsd17b4*** | L/D | 0.25544 | 1.2687 | 0.15319548 | 13h 39 min |  |
|  | 2+2+3 | 0.74814 | 1.6568 | 0.10662685 | 4h 58 min |  |
| ***Clock*** | L/D | 0.70165 | 1.04893 | 0.04199397 | 21h 18 min |  |
|  | 2+2+3 | 0.28507 | 1.24002 | 0.14058552 | 14h 00 min |  |
| ***Bmal1*** | L/D | 0.0422 | 0.92553 | 0.38969919 | 23h 00 min |  |
|  | 2+2+3 | 0.52752 | 4.15692 | 2.40881626 | 16h 46 min |  |
| ***Per1*** | L/D | 0.00551 | 1.44179 | 0.46907792 | 15h 00 min |  |
|  | 2+2+3 | 0.00006 | 1.10854 | 0.40857602 | 7h 17 min |  |
| ***Per2*** | L/D | 0.00057 | 1.32937 | 0.44962753 | 14h 12 min |  |
|  | 2+2+3 | 0.16038 | 1.16207 | 0.22779361 | 8h 57 min |  |
| ***Cry1*** | L/D | 0.00001 | 2.02643 | 0.96223012 | 14h 46 min |  |
|  | 2+2+3 | 0.20136 | 5.90168 | 1.60150356 | 11h 41 min |  |
| ***Rora*** | L/D | 0.00001 | 1.22942 | 0.25734492 | 12h 39 min |  |
|  | 2+2+3 | 0.00619 | 1.37346 | 0.29272001 | 6h 22 min |  |
| ***Rorb*** | L/D | 0.08413 | 1.14561 | 0.20535387 | 12h 25 min |  |
|  | 2+2+3 | 0.00006 | 1.26822 | 0.39069121 | 7h 46 min |  |
| ***Rev-erba*** | L/D | 0.00008 | 1.47642 | 0.80836456 | 10h 49 min |  |
|  | 2+2+3 | 0.02129 | 1.94043 | 0.6066437 | 10h 47 min |  |
| ***Rev-erbb*** | L/D | 0.0123 | 1.95588 | 0.70714853 | 13h 59 min |  |
|  | 2+2+3 | 0.61205 | 3.05012 | 0.78844209 | 18h 14 min |  |
| ***Nur77*** | L/D | 0.00003 | 1.53329 | 1.16691203 | 17h 21 min |  |
|  | 2+2+3 | 0.28743 | 1.20583 | 0.38066244 | 20h 29 min |  |
| ***Sf1*** | L/D | 0.28495 | 1.90311 | 1.03221772 | 10h 57 min |  |
|  | 2+2+3 | 0.01823 | 1.20952 | 0.10206244 | 7h 39 min |  |
| ***Creb1*** | L/D | 0.02455 | 1.1893 | 0.34177774 | 8h 10 min |  |
|  | 2+2+3 | 0.21665 | 1.07609 | 0.14729655 | 5h 11 min |  |
| **Arr19** | L/D | 0.00207 | 0.93652 | 0.2072446 | 1h 00 min |  |
|  | 2+2+3 | 0.01944 | 0.90195 | 0.24355782 | 9h 47 min |  |
| ***Dax1*** | L/D | 0.00001 | 1.54603 | 0.66703518 | 13h 48 min |  |
|  | 2+2+3 | 0.02349 | 1.512 | 0.36997332 | 16h 35 min |  |
| ***Ppargc1a*** | L/D | 0.00008 | 1.28622 | 0.3927926 | 17h 30 min |  |
|  | 2+2+3 | 0.0046 | 1.67584 | 0.48134069 | 17h 7 min |  |
| ***CytC*** | L/D | 0.0003 | 1.31791 | 0.37739491 | 18h 6 min |  |
|  | 2+2+3 | 0.00698 | 1.62506 | 0.53248387 | 17h 33 min |  |
| ***Cox4i2*** | L/D | 0.00051 | 1.59083 | 0.59334398 | 16h 9 min |  |
|  | 2+2+3 | 0.00002 | 2.09262 | 1.02718837 | 16h 42 min |  |
| ***Mtnd1*** | L/D | 0.05293 | 1.13804 | 0.23221266 | 19h 4 min |  |
|  | 2+2+3 | 0.22227 | 1.93293 | 0.45687389 | 0h 8 min |  |
| ***Tfam*** | L/D | 0.00122 | 1.23288 | 0.35812051 | 15h 3 min |  |
|  | 2+2+3 | 0.07004 | 1.30141 | 0.25030306 | 5h 52 min |  |
| ***Nrf1*** | L/D | 0.39891 | 0.96866 | 0.07605841 | 20h 25 min |  |
|  | 2+2+3 | 0.92705 | 1.16591 | 0.02950296 | 16h 18 min |  |
| ***Mfn1*** | L/D | 0.00018 | 1.00100 | 0.34526339 | 8h 6 min |  |
|  | 2+2+3 | 0.0805 | 0.98308 | 0.20086943 | 9h 40 min |  |
| ***Mfn2*** | L/D | 0.01002 | 0.88340 | 0.22406965 | 1h 30 min |  |
|  | 2+2+3 | 0.53668 | 2.29646 | 0.57474777 | 14h 30 min |  |
| ***Drp1*** | L/D | 0.0456 | 0.76137 | 0.20251469 | 0h 23 min |  |
|  | 2+2+3 | 0.13973 | 0.75654 | 0.16528663 | 5h 7 min |  |
| ***Tfeb*** | L/D | 0.26282 | 1.03071 | 0.16462686 | 9h 10 min |  |
|  | 2+2+3 | 0.2102 | 1.72723 | 0.58542092 | 15h 19 min |  |
| ***Pink1*** | L/D | 0.02403 | 2.0988 | 0.77442936 | 19h 4 min |  |
|  | 2+2+3 | 0.00175 | 2.39804 | 1.4722264 | 21h 44 min |  |
| ***Prkn*** | L/D | 0.00692 | 1.88831 | 0.97604356 | 20h 54 min |  |
|  | 2+2+3 | 0.00915 | 2.19276 | 0.47249744 | 14h 59 min |  |
| ***Opa1*** | L/D | 0.22144 | 1.05186 | 0.12222624 | 5h 10 min |  |
|  | 2+2+3 | 0.00002 | 1.03554 | 0.26308356 | 7h 59 min |  |

**ZT** is Zeitgeber time; **p** *value* - probability of outcome
