# Supplementary material for: The cost of the circadian desynchrony on the Leydig cell function
Source: Sci Rep. 2022 Sep 15;12:15520. doi: 10.1038/s41598-022-19889-9 (PMC9478133; doi:10.1038/s41598-022-19889-9)
Supplement: Supplementary file 3 — Supplementary Figure 1. [file 41598_2022_19889_MOESM3_ESM.pdf]

Supplemental Figure 1. CD altered the voluntary activity of rats.

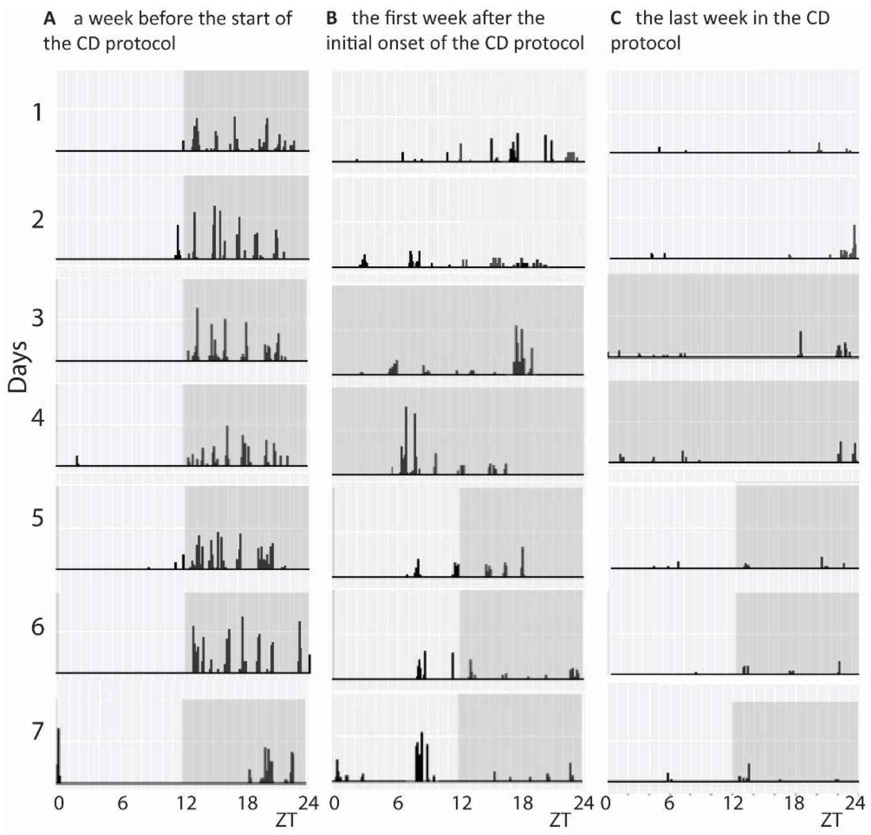

Rat voluntary activity before (A), in the first (B) and last (C) week of the two-month CD protocol.

Adult rats were housed under the controlled light regime of 12h light – 12h dark before onset of the circadian desynchrony (CD) protocol. During CD protocol, rats were exposed to a disturbing light regime (two days of continual light, two days of continual dark, and three days of 12:12h light:dark schedule) for two months. The voluntary activity was monitored, and actograms were formed. The representative weekly actogram a week before the start of the experiment (A), in the first week after the initial onset (B) and in the last week (C) of the CD protocol is shown.
